# "Mi voglio bene": a pediatrician-based randomized controlled trial for the prevention of obesity in Italian preschool children

**DOI:** 10.1186/1824-7288-36-55

**Published:** 2010-08-17

**Authors:** Paolo Brambilla, Giorgio Bedogni, Carmen Buongiovanni, Guido Brusoni, Giuseppe Di Mauro, Mario Di Pietro, Marco Giussani, Manuel Gnecchi, Lorenzo Iughetti, Paola Manzoni, Maura Sticco, Sergio Bernasconi

**Affiliations:** 1 Family pediatrician, Milano, and Department of Continuing Medical Education, Federazione Italiana Medici Pediatri, Milano, Italy; 2 Clinical Epidemiology Unit, Liver Research Center, Basovizza, Trieste, Italy and Department of Maternal and Pediatric Sciences, University of Milan, Fondazione IRCCS Cà Granda - Ospedale Maggiore Policlinico, Milan, Italy; 3 Department of Pediatrics, University Federico II, Napoli, Italy; 4 Family pediatrician, La Spezia, Italy; 5 Family pediatrician, Caserta, and Direction of Società Italiana Pediatra Preventiva e Sociale, Italy; 6 Center of Auxology and Pediatric Nutrition, Teramo, Italy; 7 Department of Continuing Medical Education, Federazione Italiana Medici Pediatri, Milano, Italy; 8 Family pediatrician, Gazzaniga, Italy; 9 Mother and Child Department, University of Modena e Reggio Emilia, Modena, Italy; 10 Family pediatrician, Robbiate, Italy; 11 Department of Pediatrics, Università di Parma, Parma, Italy

## Abstract

**Background:**

The first years of life are crucial to start preventive interventions that can have an impact on lifestyle and later overweight and obesity. Under the Italian National Health System (INHS), children are cared for by family pediatricians who perform health balances at regular intervals. The Italian Society of Preventive and Social Pediatrics (SIPPS) has designed a randomized controlled trial (RCT) to evaluate the effectiveness of family pediatricians for the prevention of childhood obesity in preschool children. We report the rationale and protocol of such trial, named the "Mi voglio bene" ("I love myself") study.

**Methods:**

"Mi voglio bene" is a parallel-arm RCT. Family pediatricians willing to participate to the trial will be randomly assigned to a control group and to an experimental group. The control group will provide the usual standard of care while the experimental group will implement 10 preventive actions (promotion of breastfeeding, avoidance of solid foods, control of protein intake, avoidance of sugar-sweetened beverages, avoidance of bottle, active means of transportation, identification of early adiposity rebound, limitation of television viewing, promotion of movement, and teaching portion size) at 10 time points during a 6-yr follow-up. The main outcome measures is the prevalence of overweight and obesity at 6 years of age. The experimental intervention is expected to reduce the prevalence of overweight and obesity from 25% to 20% and the study requires a total of 3610 children. Each pediatrician will enroll 30 consecutive newborns into the study so that a total of 120 pediatricians will participate to the study.

**Discussion:**

"Mi voglio bene" is expected to provide important information for the INHS and possibly other institutional child care settings about the effectiveness of a pediatrician-based approach to the prevention of childhood obesity. We published this study protocol with the aim of opening a discussion with all people interested in fighting childhood obesity and to receive useful criticisms.

## Background

The first years of life are crucial to start preventive interventions that can have an impact on lifestyle and later overweight and obesity [1]. Accordingly, preventive interventions focusing on children between the ages of birth and 5 years are gaining increasing attention from researchers [1,2].

A recent systematic review identified 7 randomized controlled trials (RCT) aimed at preventing obesity in preschool children [1]. Four trials were carried out in preschool setting, 2 were family-based and 1 was carried out in maternity hospitals by promoting breastfeeding. None of these trials had an effect in preventing overweight or obesity. Most of these trials had BMI as outcome measure but centile crossing or the timing of adiposity rebound may be better indicators of growth exceeding the expected standards in children under 5 years of age [1]. Another reason why these trials have given negative results might be that the interventions were not compulsory or not given for enough time [1].

Another recent systematic review identified 23 randomized and non-randomized clinical trials aiming to prevent obesity in preschool children [2]. Most of these studies were conducted in either the home or preschool/child care setting and interventions designed to impact not only on knowledge but also on skills where somewhat more effective [2]. The involvement of parents was identified as essential to make children receptive to prevention programs [2].

The prevalence of overweight and obesity in Italian preschool children is high as in many Western countries [3]. Under the Italian National Health System (INHS), children are cared for by family pediatricians, who perform health balances (HB) at regular intervals. The Obesity Working Group of the Italian Society of Preventive and Social Pediatrics (SIPPS) has designed a RCT to evaluate the effectiveness of family pediatricians for the prevention of childhood obesity in preschool children. In this paper we report the rationale and protocol of such trial, named the "Mi voglio bene" ("I love myself") study.

## Methods

### Study design

"Mi voglio bene" is a parallel-arm RCT. Family pediatricians willing to participate to the trial will be recruited through advertisements on scientific journals and websites or direct contact at scientific meetings. They will be randomly assigned to a control group and to an experimental group. The control group will provide the usual standard of care while the experimental group will implement the 10 preventive actions that make up the experimental intervention (promotion of breastfeeding, avoidance of solid foods, control of protein intake, avoidance of sugar-sweetened beverages, avoidance of bottle, active means of transportation, identification of early adiposity rebound, limitation of television viewing, promotion of movement, and teaching portion size; see below for details). Such actions will be performed at 10 time points corresponding to HB performed for INHS (1.0 to 1.5 months, 2.5 to 3.0 months, 5.0 to 6.0 months, 8.0 to 9.0 months, 11.0 to 12.0 months, 16.0 to 18.0 months, 24.0 to 30.0 months, 36.0 to 42.0 months, 48.0 to 54.0 months and 66.0 to 72.0 months; see below for details). Blinding and concealment are not possible owing to the study design. Written informed consent will be obtained from the legal guardians of the children and the study protocol will be submitted to an Ethics Committee for approval. The trial will be registered on the ISRCT register http://www.isrctn.org.

### Main outcome

We hypothesize that the 10 preventive actions will reduce the prevalence of overweight and obesity at 6 years of age from 25% to 20%. In order to detect this difference as statistically significant at an alpha level of 0.05 with a power of 90%, 1504 children per group are needed. This number will be increased to 1805 children per group owing to an expected drop-out of 20% during the study. Thus, a total of 3610 children will be required for the study. Because each study pediatrician is expected to enroll 30 consecutive newborns, a total of 120 pediatricians will participate to the study. Presence of chronic disease requiring specific counseling on nutrition or lack of command of the Italian language will be reasons for exclusion.

### Secondary outcomes

The availability of 10 time points from the age of birth to 6 years is a great strength of the trial. We plan to use these time points to compare trajectories of BMI, BMI centile crossing, and adiposity rebound in the two groups using mixed models [4,5].

### Measurements and data entry

The reported compliance of the children with the 10 preventive interventions will be recorded during the study. Weight, length (≤ 24 months of age) and height (> 24 months of age) will be measured following the Anthropometric Standardization Reference Manual [6]. Overweight and obesity will be diagnosed using the criteria put forth by the International Obesity Task Force [7]. The early adiposity rebound will be defined as proposed by Rolland-Cachera [8] and patterns of BMI centile changes will be analyzed as suggested by Cole [5]. Waist circumference [9] and blood pressure [10] will also be measured at the 10^th ^HB (66.0 to 72.0 months). Web-based data entry will be performed in respect of Italian privacy legislation using the services of a commercial provider (Medidata, Modena, Italy).

### Experimental intervention

The 10 preventive interventions are listed in Table 1 and described in detail below.

**Table 1:** The 10 preventive actions and their timing during the 6-year follow-up.

	Health balance Months									
	**1**	**2**	**3**	**4**	**5**	**6**	**7**	**8**	**9**	**10**

	**1.0-1.5**	**2.5-3.0**	**5.0-6.0**	**8.0-9.0**	**11.0-12.0**	**16.0-18.0**	**24.0-30.0**	**36.0-42.0**	**48.0-54.0**	**66.0-72.0**

Breastfeeding	X	X	X							

Avoidance of solid foods	X	X	X							

Control of protein intake	X	X	X	X	X	X	X			

Avoidance of sugar-sweetened beverages	X	X	X	X	X	X	X	X	X	X

Avoidance of bottle						X	X			

Means of transportation					X	X	X	X	X	X

Early adiposity rebound							X	X	X	X

Television viewing					X	X	X	X	X	X

Movement and active play					X	X	X	X	X	X

Control of portion size							X	X	X	X

#### Action 1 - Breastfeeding during the first 6 months

While many cohort studies suggest that breastfeeding may protect from obesity [11], the few available RCT do not support this conclusion [1]. Mothers will be asked to breastfed their children for at least 6 months following the general recommendation of the European Society of Pediatric Gastroenterology, Hepatology and Nutrition (ESPGHAN) [12]. This action will be initiated at the 1^st ^and continued up to the 3^rd ^HB. (Mothers willing to breastfed their children up to 1 year of age will be encouraged to do so [12].)

#### Action 2 - Avoidance of solid foods during the first 6 months

Evidence from cohort studies suggests that the avoidance of solid foods during the first months of life may protect from obesity [13,14]. The introduction of solid foods and caloric beverages will be discouraged during the first 6 months of life unless suggested by special needs, following the general recommendation of ESPGHAN [12]. This action will be initiated at the 1^st ^and continued up to the 3^rd ^HB.

#### Action 3 - Control of protein intake during the first 24 months

A recent RCT has provided evidence that a lower protein intake during the first 2 years of life may protect from obesity [15]. Protein intake will be kept low by giving simple indications on foods to introduce on the basis of the milk consumed (Table 2). In detail, there will be no limitation to the consumption of human milk. For formula-fed infants, low-protein formulas will be suggested and upper limits of intake will be given. Toddler milk will be recommended in the second year of life. In order to make recommendations as practical as possible, limits of consumption will be given only for meat, jam, cheese, fish, and yogurt. This action will be initiated at the 1^st ^and continued up to the 7^th ^HB.

**Table 2:** Suggested strategies to control protein intake during the first 24 months.

	Human milk	Formula milk	Toddler milk	Cow milk
0-6 months	No limitation	Up to 1000 mL/day (at 6 months) with low-protein formula	Not allowed	Not allowed

6-12 months	No limitation	Up to 500 mL/day with low-protein formula	Not allowed	Not allowed
	Meat = 30 g	Meat = 30 g		
	Jam = 30 g	Jam = 30 g		
	Cheese = 20 g	Cheese = 20 g		
	Parmesan = 1 little spoon	Parmesan = 1 little spoon		
	Yogurt = 60 g	Yogurt = 60 g		

12-24 months	No limitation	Not allowed	Up to 500 mL/day with low-protein formula	Up to 300 mL/day
	Meat = 30 g		Meat = 30 g	Meat = 30 g
	Jam = 30 g		Jam = 30 g	Jam = 30 g
	Cheese = 20 g		Cheese = 20 g	Cheese = 20 g
	Parmesan = 1 little spoon		Parmesan = 1 little spoon	Parmesan = 1 little spoon
	Yogurt = 60 g		Yogurt = 60 g	Yogurt = 60 g

#### Action 4 - Avoidance of sugar-sweetened beverages during all the study

Many cohort studies and some school-based RCT have shown that the consumption of sugar-sweetened beverages is a risk factor for obesity [16,17]. The consumption of sugar-sweetened beverages will be discouraged during all the study unless suggested by special needs. Only milk and plain water will be allowed. This action will be initiated at the 1^st ^and continued up to the 10^th ^HB.

#### Action 5 - Avoidance of bottle after the first 24 months

Bottle use is emerging as risk factor for childhood obesity independently from the kind of beverage and its energy content [18]. A very recent RCT has shown that an office-based intervention may decrease the use of bottle [19] but the effect on prevention of obesity is not known. This action will be initiated at the 6^th ^and continued up to the 7^th ^HB.

#### Action 6 - Means of transportation

Although the RCT performed so far in preschool children have given negative results [1], physical activity is a mainstay of the prevention of obesity. Use of electric toys such as cars and motorcycles will be discouraged from 1 year of life. Use of baby trolleys will be discouraged from 3 years of life. This action will be initiated at the 5^th ^and continued up to the 10^th ^HB.

#### Action 7 - Early adiposity rebound

The adiposity rebound, defined as the second rise in BMI between 3 and 6 years of age, is a risk factor for later obesity [5,8]. Starting from the 7^th ^HB, the study pediatricians will systematically evaluate and record whether there has been an adiposity rebound. The presence of such rebound will serve to reinforce the message on nutrition and lifestyle changes.

#### Action 8 - Limit television viewing

Television viewing is an accepted risk factor for childhood obesity [20]. A recent RCT showed a reduction in sedentary behavior and energy intake stemming from reduced TV viewing [21]. TV viewing will be discouraged up to 2 years of age and limited to 8 hours per week thereafter. This action will start at the 5^th ^HB and continue up to the 10^th ^HB as TV viewing has been reported in Italian children as young as 1 year (unpublished data).

#### Action 9 - Movement and active play

As noted under *Action 6*, exercise is a mainstay of the prevention of obesity. The study pediatricians will suggest which active plays should be performed by the children in relation to their age. Preference will be given to activities that can be done together with parents and peers. This action will start at the 5^th ^HB and continue up to the 10^th ^HB.

#### Action 10 - Control of portion size

Teaching control of portion size may be useful to prevent obesity [22,23]. We plan to develop a Photographic Atlas of Food Portion Sizes specific for Italian children similarly to what has been done for Italian adults [24]. This action will start at the 7^th ^HB and continue up to the 10^th ^HB.

## Conclusion

We discussed the rationale and the protocol of the "Mi voglio bene" study, a pediatrician-based RCT for the prevention of obesity in Italian preschool children. Our study differs from the presently available RCT for three main reasons [1]: 1) it is pediatrician-based, a circumstance made possible by the fact that under INHS children are cared for by family pediatricians; 2) it is cost-effective, as it will require only a minimal increase of the time needed to perform already scheduled HB; 3) the availability of many (10) encounters will allow to reinforce the preventive messages. We expect that this trial will provide important information for the INHS and possibly other institutional child care settings about the effectiveness of a pediatrician-based approach to the prevention of childhood obesity.

## Abbreviations

ESPGHAN: European Society of Pediatric Gastroenterology, Hepatology and Nutrition; HB: health balance; INHS: Italian National Health System; RCT: Randomized controlled trial; SIPPS: Società Italiana di Pediatria Preventiva e Sociale.

## Competing interests

The authors declare that they have no competing interests.

## Authors' contributions

All the Authors contributed to the development of study protocol. The manuscript was draft by PB, revised by GB, and read and approved by all Authors.
